# A novel curricular framework to develop grant writing skills among MD–PhD students

**DOI:** 10.1017/cts.2022.384

**Published:** 2022-04-04

**Authors:** Jaclyn P. Souder, Mark E. Pepin, Randy L. Seay, Robin G. Lorenz, William M. Geisler, Talene Yacoubian

**Affiliations:** 1 Medical Scientist Training Program, Heersink School of Medicine, University of Alabama at Birmingham, Birmingham, AL, USA; 2 Department of Research Pathology, Genentech Inc, South San Francisco, CA, USA

**Keywords:** Physician-scientist, training grant, mentorship, career development

## Abstract

**Background/Objectives:**

Physician-scientists have long been in high demand owing to their role as key drivers of biomedical innovation, but their dwindling prevalence in research and medical communities threatens ongoing progress. As the principal avenue for physician-scientist development, combined MD–PhD training programs and NIH-funded Medical Scientist Training Programs (MSTPs) must address all aspects of career development, including grant writing skills.

**Methods:**

The NIH F-series grants – the F30 grant in particular – model the NIH format of federal funding, and are thus ideal opportunities to acquire biomedical research grant preparation experience. Therefore, in this report, we describe a curricular model through which predoctoral MSTP students obtain exposure to – and training for – F-series grant conceptualization, writing, and evaluation.

**Results:**

Since the development of these longitudinal courses, we observed trending improvements in student funding success rates, particularly among original submissions, and perceived benefits among participating students.

## Introduction

Physician-scientists deliver irreplaceable value to both clinical and scientific communities by bridging efforts to treat both human disease and innovate healthcare delivery. A central “pipeline” for training physician-scientists is the dual-degree (MD–PhD) training program, which provides an integrated predoctoral training framework, linking MD and PhD training for trainees with potential as leaders in both biomedical research and clinical practice [1]. Among the basic skills needed for a successful career, securing extramural funding is paramount, whereby acquisition of research materials, personnel, and protected time permit independent research [1,2]. Unfortunately, MD–PhD trainees experience a longer time between degree(s) completion and first R-series grant awarded [3,4], and national survey-based evidence suggests that MD–PhD graduates achieve equivalent funding rates to their MD-only colleagues [3]. Therefore, MD–PhD training should better address the need to equip MD trainees with a curriculum of grant writing instruction.

Barriers to grant funding success are far reaching and include lengthy periods of clinical training, and reduced research time, inadequate mentorship, and lack of institutional support [4]. While many of these barriers may be addressed by having dedicated hands-on training to develop grant writing skills [5], no courses have been reported to develop this skill for physician-scientists at the predoctoral stage of training. At the postgraduate level, one course for clinical and postdoctoral psychiatry fellows consists of 25 sessions held annually [6], the ultimate goal of which is to facilitate application for career development (K-series) awards. Another reported course, offered to biomedical predoctoral (PhD) trainees for F31 preparation through weekly meetings, is held during the second year of PhD training [7]. Despite the reported advantages of these preparatory courses in improving grant funding success, no such course has been described for MD–PhD trainees.

Therefore, the current document outlines a two-tiered curricular approach to grant writing preparedness for MD–PhD students. We propose that predoctoral grant writing training in grant writing would likely improve long-term retention and success of physician-scientists, bolstering this dwindling workforce in the academic sector.

## Methods

### Course structure: *Survival Skills for Physician-Scientists*

The NIH-funded Medical Scientist Training Program (MSTP) at the University of Alabama at Birmingham (UAB) has taken multiple steps to encourage and develop grant writing skills in MSTP students. Beginning with the entering class of 2013, the MSTP instituted the formal requirement for all students to submit an F-series grant (F30/F31). In accordance with NIH guidelines, students were required to submit within 4 years of matriculation (year 2 of graduate research).

In 2014, a course entitled *Survival Skills for Physician Scientists* (STP2043) was created in the summer between the first and second year of preclinical medical training, a time at which time all trainees complete their second research laboratory rotations. The overall goal of this course is to introduce students to the grant writing process, give them a more involved and deeper understanding of their rotation projects, and facilitate general career development skills. To accomplish this, students are introduced to all components of an NIH training grant, and they practice writing scientific “specific aims” and training plans based on their summer research project; this is done in close collaboration with their summer research supervisors. Weekly meetings last 60–90 min consist of an overview of funding basics, career development, interpersonal interactions, time and data management, overview and completion of a Specific Aims page and Research Training Plan. Assigned resources given to all students include *Making the Right Moves: A Practical Guide to Scientific Management for Postdocs and New Faculty* [8], *Mastering Your PhD: Survival and Success in the Doctoral Years and Beyond* [9], *Grant-writing Instructions*, *A Practical Guide to Writing a Ruth L. Kirchstein NRSA Grant* [10], and *The Grant Application Writer’s Workbook* [11]. The last – and most positively received – element of this course is a mock Study Section, wherein students are assigned to evaluate grants written by other students in their cohort. The final meeting consists of an in-person, student-led study section to discuss strengths and scoring of each grant.

The *Survival Skills for Physician Scientists* course was reorganized in 2019 around topics including an introduction to fellowship grants, review of example F30 grant applications, authorship, a literature review with a UAB-affiliated librarian, an overview of the Background and Goals section, Specific Aims, and Research Training plan, a mentorship lecture with the graduate school dean, introduction to Individual Development Plan, and a Mock Study Section for peer and faculty review of Specific Aims pages at the end of the course. These changes were designed to provide students with a broad understanding of grant writing, the review process, and to focus on those aspects of grant writing that do not necessarily require a fundable project. The writing assignments were targeted to those sections describing their scientific history and career goals (“Biosketch” and the “Background/Goals” section) and on the Aims page. Key career development principles relevant to grant writing, such as literature review and mentorship, were also added, while topics such as time and data management and interpersonal interactions were limited to reading assignments and brief discussions.

### Course structure: *Grants & Grubs*

An additional series of lunch lectures, entitled *Grants & Grubs*, was constructed to help trainees prepare sections of their grants during the process of their F-series grant-writing. As such, the goal of this course was to assist with the creation and submission of an F30 or equivalent grant application. The monthly series consists of 7 lectures (1.5 h/session) offered during the first and second years of PhD graduate research. Alongside MSTP faculty/administration, all sessions are co-led by MSTP students who had already previously submitted – and subsequently been awarded – an F-series grant. Lectures provide an overview of the different grant components as well as presentation of the other more technical components of the grant including forms, approvals, and letters of recommendation and support. Students are not required to write the components of the grant as part of the course, but can bring their grants for review prior to submission deadlines for real-time feedback. Resources included *Making the Right Moves* [8], *The Best Kept Secrets to Winning Grants* [12], *Grant Application Writer’s Workbook* [11], *A Practical Guide to Writing a Ruth L. Kirchstein NRSA Grant* [10], numerous internal F30/F31 examples, and “LOR Advice.” To encourage student participation and active learning, lectures were interactive with assigned readings from *A Practical Guide to Writing a Ruth Kirschstein NRSA Grant* [10], along with *The Grant Application Writer’s Workbook* [11]. Based on student feedback, required homework assignments beginning in 2020 consisted of grant review of provided sample grants and drafting various elements of an F-style grant (Biosketch, Background and Goals, Specific Aims, Research Strategy) with a Mock Study Section for peer and faculty review at the culmination of the course. To provide practical assistance with grant logistics, speakers from outside the UAB MSTP program were invited to discuss topics such as the Office of Sponsored Programs and IACUC/IRB approval. Other course lectures were taught by UAB MSTP faculty. The F30 grant application assignments during this served as an initial draft that the students could then modify over the course of the next year to fulfill the F30/F31 grant submission requirement for students prior to the end of their second year in the PhD phase (GS2 year, 48 months into training). A sample schedule for the course is provided in Table 1.

**Table 1.** Modified course structure for *Grants & Grubs*

Lecture number	Presenter	Topic/Content	Required Reading (Hollenbach [10])	Required homework
1	UAB MSTP Director; more senior MSTP students	Fellowship Grant Introduction; Student perspective on submitting F grants	Ch. 2 – The People Behind the Curtain (Review Process)	Review example grants
2	UAB CCTS Training Academy; MSTP Director	UAB resources for submitting grants; Discuss example grants; Writing a Biosketch	n/a	Complete draft of Biosketch
3	UAB Office of Sponsored Programs; MSTP Director	Introduction to the Office of Sponsored Programs and Applicant-specific Information; Review of Biosketch drafts; Writing the Background & Goals section	Ch. 3 – Who are you? Ch. 4 – Who’s your boss?	Complete draft of Background & Goals
4	UAB MSTP Director	Research Training Plan: Aims and Research Strategy; Review of Background & Goals drafts; Writing the Institutional sections; RCR	Ch. 5 – Blind them with science Ch. 7 – Details, details, details	Complete draft of Specific Aims, begin Research Strategy
5	UAB IACUC and MSTP Director	Introduction to IACUC; Vertebrate Animal Section; Review of Specific Aims drafts	n/a	Continue draft of Research Strategy
6	UAB IRB Staff and MSTP Director	Overview of IRB protocols; Human Research Subject section	n/a	Continue draft of Research Strategy
7	UAB Grants Administrator; MSTP Director	After your grant is awarded: Just in Time and research performance progress report; Review process and Study Section	Ch. 8 – Now what?	Complete draft of Research Strategy
8	n/a	Mock Study Section	n/a	n/a

CCTS, Center for Clinical and Translational Science; MSTP, Medical Scientist Training Program; IRB, Institutional Review Board; IACUC, Institutional Animal Care and Use Committee.

### Course evaluations

Evaluations for the *Survival Skills for Physician Scientists* and *Grants & Grubs* courses were collected from students via online forms, given anonymously. A 5-point Likert agreeability scale ranging from "Strongly Disagree” to "Strongly Agree” was used to determine student satisfaction with different aspects of the course. Responses from two to nine students were analyzed for each course per year from 2014 to 2019 for *Survival Skills for Physician Scientists* and from 2019 to 2020 for *Grants & Grubs*. The total number of responses collected and pooled for each course ranged from 7 to 40. Data were reported as frequency of responses (percentage of respondents selecting each point on the scale). Ethical approval was provided by the UAB Office of the IRB.

### Calculation of funding rates

Data on F30/F31 funding rates for MD–PhD students and other graduate-level students at UAB from 2013 to 2021 were provided by the UAB Office of Sponsored Programs. The start year used was defined as the actual or anticipated year that funding would begin, rather than the year the grant was submitted. Data used for analyses included student program (MSTP Graduate Biomedical Sciences (GBS) doctoral program), activity description (F30 or F31), sponsor within NIH, grant status (active, completed, or not funded), type of submission (original, i.e., new, or resubmission), and dates of award. Withdrawn applications or those transferred from another institution were excluded from analysis, and all award information was de-identified. National success rates were obtained from NIH RePORT (report.nih.gov). It should be noted that for UAB and GBS we collected data on all submissions (whether reviewed or not), while for NIH the only data reported were for applications that were reviewed. Data on the number of grant submissions at UAB prior to 2013 could not be obtained so success rates for years prior to 2013 are not reported. We calculated the number of grant submissions, number of grants awarded, and the percent funded/success rate (number of grants awarded/number of submissions × 100) for each group from 2013 to 2019. Data for the MSTP F-series grants were pooled due to the comparatively low number of submissions compared to GBS and national submissions.

### Statistics

Success rates were compared between different groups using Student’s unpaired t test for comparisons of two groups, and False Discovery Rate two-stage step-up method of Benjamini, Krieger, and Yekutieli was used when multiple t-tests were performed. Success rates over time by year were analyzed using simple linear regression, with statistics computed relative to a horizontal slope. Significance was defined as *p*-value less than 0.05. All statistical calculations were performed using GraphPad Prism v 9.1.0.

## Results

### Course evaluations

**Fig. 1.**
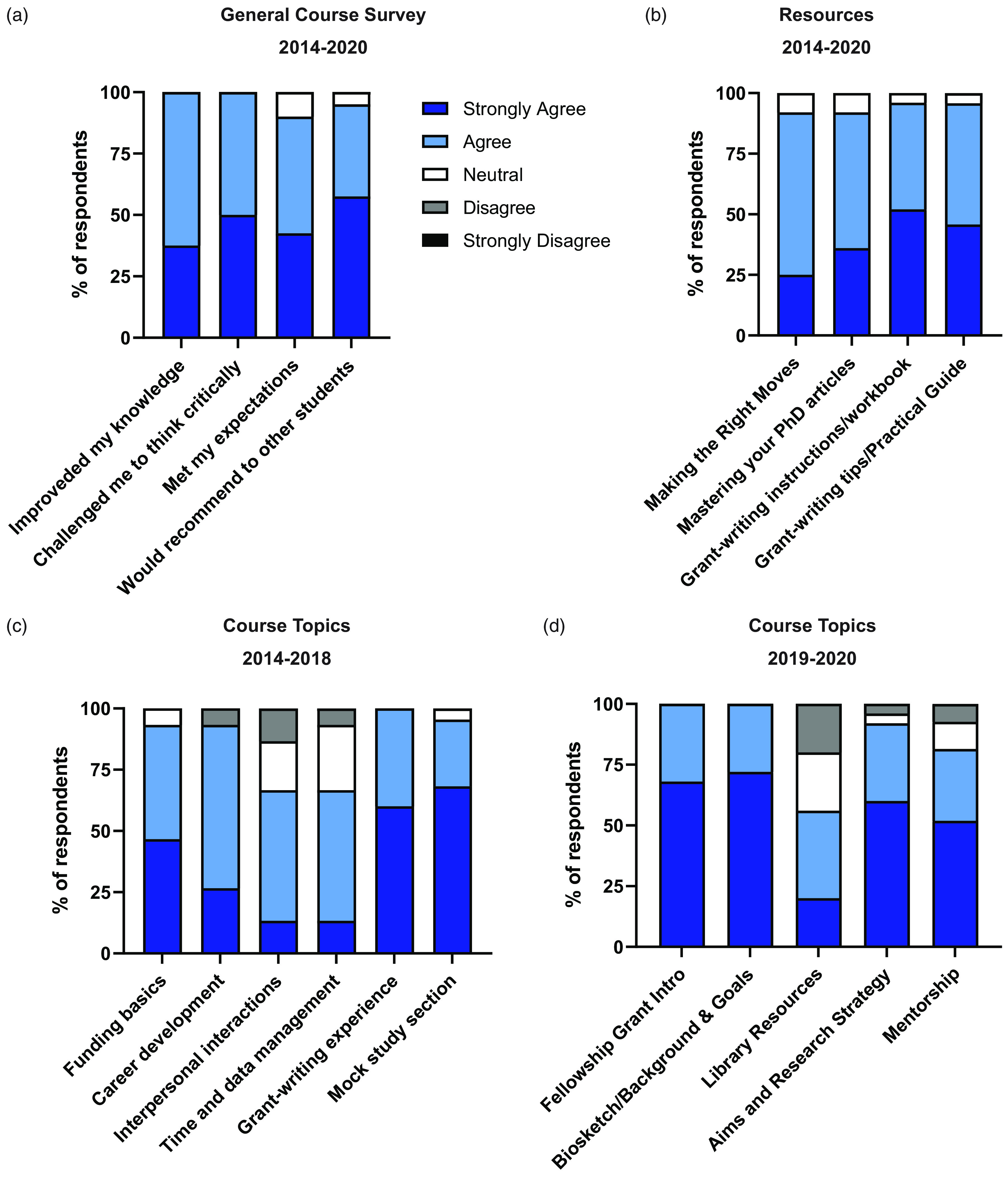
Course evaluations for *Survival Skills for Physician Scientists.* Anonymous survey responses collected from students anonymously online using a 5-point Likert scale from “Strongly Agree” to “Strongly Disagree.” (a) Overall appraisal of course benefits by participating students. (b) Suitability of course materials and resources made available during the course. (c) Feedback form students regarding course lecture content.

**Fig. 2.**
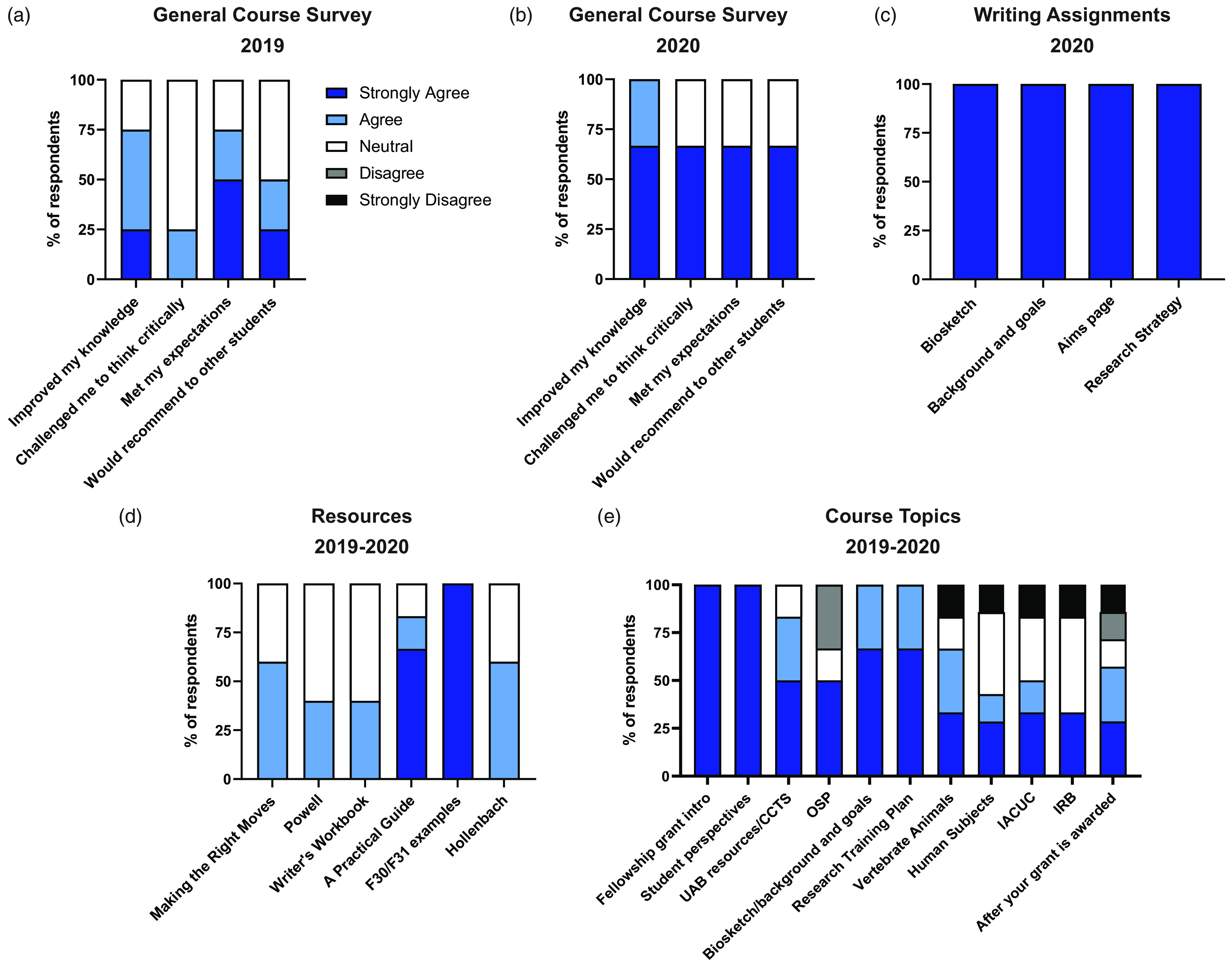
Course evaluations for *Grants & Grubs.* Anonymous survey responses collected from students anonymously online using a 5-point Likert scale from “Strongly Agree” to “Strongly Disagree.” The overall impression by students following (a) year 1 (b) and year 2 of course offering. (c) Feedback of the writing assignments for year 2. (d) Feedback based on course materials and resources available. (e) Feedback form students regarding course lecture content.

To monitor student satisfaction and perceived effect on readiness for grant submission, we analyzed course evaluations from both the *Survival Skills for Physician Scientists* and *Grants & Grubs* courses. Overall, we found that students were satisfied with the course structure and would recommend these courses for other students (Figs. 1 and 2). Feedback for *Survival Skills for Physician Scientists* was generally positive with 100% of survey respondents reporting that they agree or strongly agree that the course improved their knowledge and challenged them to think critically (Fig. 1a). All resources used in the course were positively favored by student respondents (Fig. 1b). For topics provided in the course from 2014 to 2018, those with the most positive feedback included “Funding basics,” “Grant-writing experience,” and “Mock Study Section” (Fig. 1c). The topics with the most negative feedback (“Interpersonal Interactions” and “Time and Data Management”) were excluded from the course in subsequent years. Following adjustment of the course curriculum in 2019, the topics of greatest perceived benefit with 100% of respondents who agreed or strongly agreed that content was appropriate for the course and should be kept as part of the curriculum were the “Fellowship Grant Introduction” and “F-grants, Biosketch, Background and Goals” (Fig. 1d). When asked to identify the best timing for the *Survival Skills for Physician Scientists* course, 100% of survey respondents preferred hosting the course between the first and second years of medical school. Free-response comments from students focused largely on lecture content, writing assignments, and the peer review component of the course. Additional topics students suggested for inclusion in the course include a discussion of preliminary data and when to include, where to find funding opportunities, and overcoming “imposter syndrome.” Other free-response comments highlighted the usefulness of peer mentorship, structured planning, and even confidence building.

Overall feedback for the *Grants & Grubs* course was also generally positive, with 85.7% of respondents agreeing that the course improved knowledge overall (Fig. 2a–c). Among the materials provided, previously-funded F30/F31 grants were deemed most valued by 100% respondents, with *A Practical Guide* (83.3%) and *Making the Right Moves* (60%) printed materials receiving positive feedback(Fig. 2d). The most highly-regarded course topics emphasized writing and analyzing F-series grant sections (Fig. 2e). During the last year of reporting, grant section drafting became a required assignment; at which time 3/3 (100%) of respondents found Biosketch, Background & Goals, Specific Aims pages, and Research Strategy to be helpful aspects of course lecturing (Fig. 2f). Additional free-response comments highlighted the need to further condense IACUC/IRB/OSP lectures, request for individualized feedback, and supported the practice of drafting grant sections.

### Success rates

**Fig. 3.**
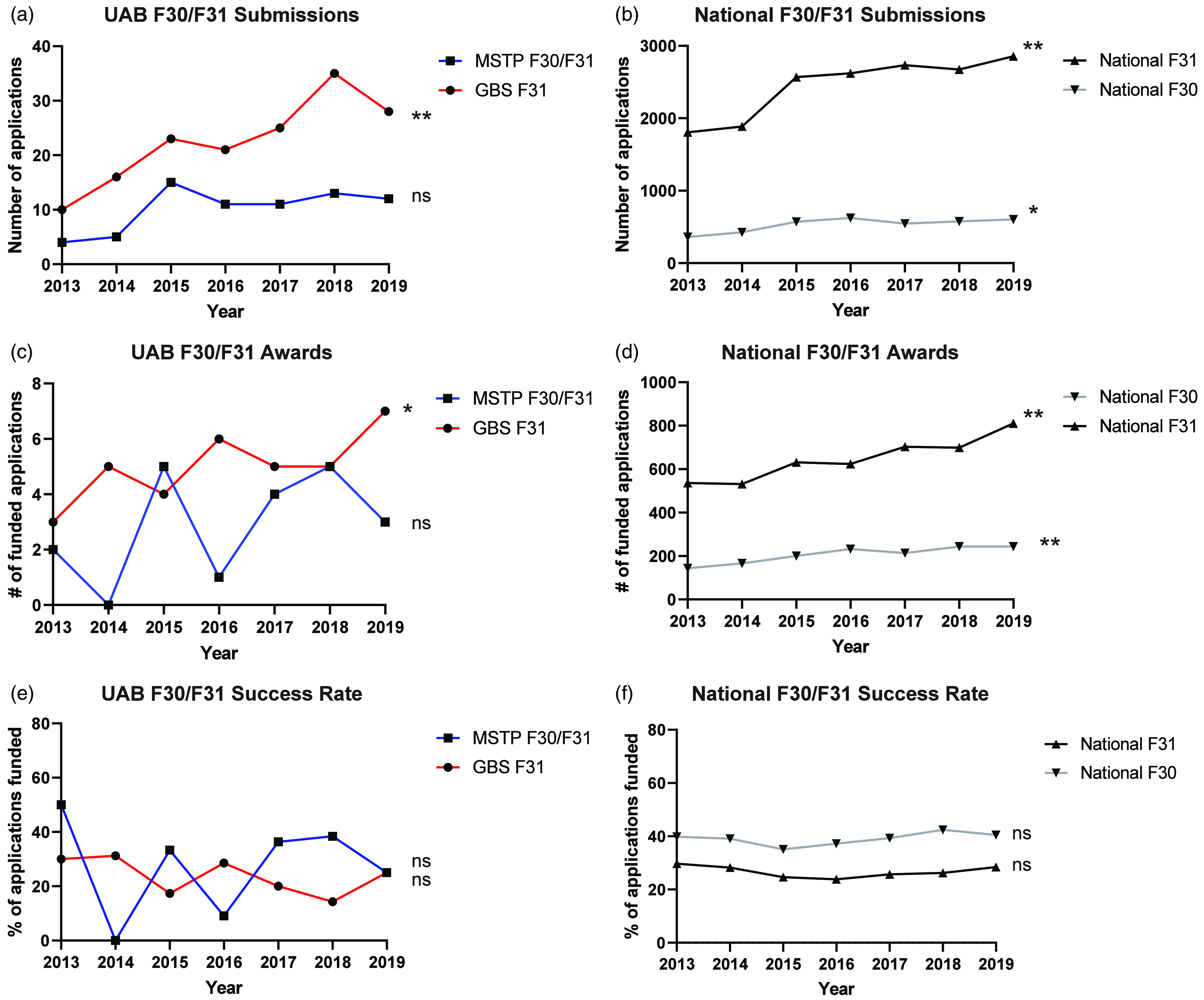
F-series grant submissions and funding rates. Using data from the UAB Office of Sponsored Programs and NIH RePORT (report.nih.gov), student grants Figure legend in A applies to D and G, figure legend in B applies to E and H, figure legend in C applies to F and I. The number of F-series applications submitted among (a) UAB graduate biomedical science PhD (GBS) and MD-PhD (MSTP) students and (b) national F30/F31 submissions. The number of F-series awards granted for (c) UAB GBS and MSTP and (d) national F30/F31 funding. The percentage of funded grants for (e) UAB GBS and MSTP and (f) national F30/F31 rates. All rates analyzed for each group with simple linear regression, significance was calculated based on probability of having a null slope with *p < 0.05, **p < 0.01, ***p < 0.001, ****p < 0.0001, GBS, Graduate Biomedical Sciences; MSTP, Medical Scientist Training Program; ns, not significant; UAB, University of Alabama at Birmingham.

To begin to understand the effect of compulsory grant submission and formalized grant writing training on F-series funding success rates, cohort outcomes were compared with comparable F-series (F31) grant funding rates from both the GBS program and national F-series funding rates. In accordance with the new requirement, MSTP student F30/F31 submissions sharply increased threefold and remained stable until the end of reporting period (Fig. 3a). Although the number of both submissions and funded grants also gradually increased among GBS students (Fig. 3c, *R* ^2^ = 0.80, *p* = 0.01), the proportion of funded grants remained unchanged (Fig. 3e). Similarly, the national rates of submitted (Fig. 3b) and funded (Fig. 3d) F-series grants increased, whereas the national funding proportion remained unchanged (Fig. 3f). Although MSTP grant submission dynamics reflect similar trends, no significant inferences could be made owing to low cohort sample sizes.

**Fig. 4.**
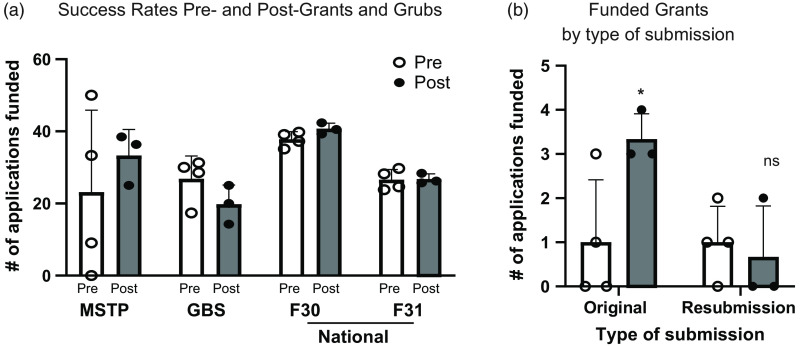
UAB MSTP F-series success rates. The funding outcomes grants were compared pre- (unfilled circles) and post- (black circles) implementation of the *Grants & Grubs* course. (a) Average funding rates for the UAB MSTP combined F-series grants, GBS F31 grants, and National F-series rates pre- and post-implementation of the *Grants & Grubs* course. The (b) Number and (c) percentage of funded F30/F31 grants submitted by the UAB MSTP as either original or resubmissions pre- and post-course. Groups for each graph (a–c) were compared using multiple unpaired t tests, where significance was designated as *p < 0.05, GBS, Graduate Biomedical Sciences; MSTP, Medical Scientist Training Program; ns, not significant; UAB, University of Alabama at Birmingham.

To begin to understand whether participation in the *Grants & Grubs* course influenced grant funding outcomes, we pooled success rates from 3 years before and after its institution. Although the overall percentage of funded grants remained unchanged (Fig. 4a), we noticed a significant increase in the number of funded MSTP submissions among original submissions (Fig. 4b).

**Fig. 5.**
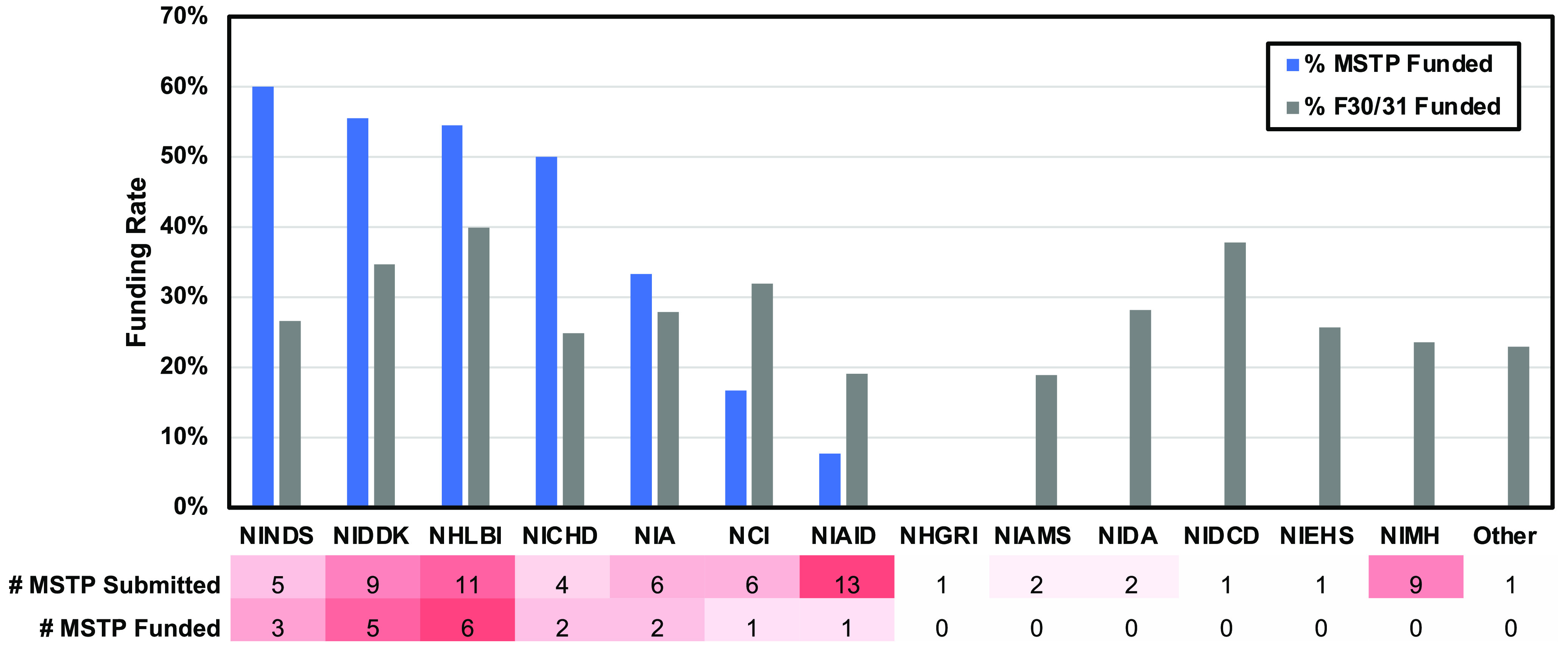
Distribution of submissions and funded awards among NIH institutes. Percentage of MSTP student funding (blue) relative to national funding rate (gray) for NIH institutes offering F-series grants during the course implementation period. Below, a table illustrates the total number of MSTP trainees that submitted and received fundable scores to each institute. MSTP, Medical Scientist Training Program.

To compare the performance of our MSTP students with national trends in F-series grant funding, the funding rates of our cohorts were compared with national funding trends over the same time interval (2013–2019) (Fig. 5). We found that MSTP students had a higher percentage of F-series grant funding compared with national averages among the institutes to which a majority of MSTP student submitted. By contrast, institutes to which very few (<4) MSTP students submitted were never funded. Although further studies are warranted, these data may support – among other possibilities – that vertical support mechanisms of grant writing (e.g., examples of funded F30/31 grants, experienced mentorship, and so forth…) improves the probability of subsequent funding.

## Discussion and conclusions

In the current report, we provide a novel framework for developing MD–PhD student grant writing skills, using F-series grants as mechanisms of both financial support and growth through a necessary career skill. Implementing similar training support for MSTP trainees will likely improve trainee preparedness in other domains, bolstering a healthcare workforce that is essential for medical innovation. Overall, we believe that predoctoral grant writing coursework may improve success in both retention and success of academic physician-scientists academia.

Despite its benefit to trainees, the incorporation of grant writing training is largely lacking among current MD–PhD – including NIH MSTP – programs. According to the information provided on national MSTP websites, only 44% (22/50) of current NIH-funded MSTP programs provide grant writing preparation/training, and fewer (18%, 9/50) offer dedicated courses and/or mock study sessions. Although 44% of programs endorse student applications for extramural funding, only 7/50 programs require grant submission as a component of training. Therefore, although several institutions provide longitudinal grant writing support to trainees, our observations suggest that the grant writing preparation varies widely across institutions.

However, we did observe a more consistent funding rate and number of awards with reduced variability for F30 grants, particularly original submissions, by our UAB MSTP students following initiation of the *Grants & Grubs* course from 2017 to 2019, suggesting that students are submitting fundable applications more consistently. Additionally, it should be noted that seeing the effect of recent course changes will take up to 2 years to be apparent. We also looked at the distribution of funded grants by institute for both UAB programs and compared this to the national distribution of funded grants by institute. While we expected the number of institutes applied to for UAB MSTP and GBS students to be lower than that for national F-series submissions, the number of institutes for funded grants from UAB students was much shorter, with only 7/13 total institutes funding UAB MSTP awards. Further, the top NIH institute for each program funded 30% of UAB MSTP awards (NHLBI), whereas the top national rate of 26.8% originated from the NCI.

Aside from increasing success rates for F-series grants, a more intangible desire of the course was to foster students’ confidence in grant writing and submissions. We consistently noted from subjective evaluations that students were challenged by our courses, improving knowledge through hands-on practice. Specifically, our students directed most positive feedback to hands-on activities within the course where they could receive feedback on their projects and writing style via peer reviews. Students also noted that access to a database of recently funded F30/F31 grants serves as a central resource during their drafting process. Course timing was also identified as an important indicator of usefulness, especially for *Grants & Grubs*. Students enjoy participating in a less-intensive course during the preclinical stage, or the summer spanning the first 2 years of medical school. Similarly, trainees felt that beginning the *Grants & Grubs* course in spring of their first year was early enough in the PhD to give adequate time prior to grant submission, while still allowing sufficient time to conceptualize their projects.

Despite the benefits observed among trainees in response to our course offerings, a few limitations in our analysis should be considered. Firstly, our single-center cohort study design precludes us from making broad conclusions regarding course outcomes. Furthermore, measuring the effectiveness of curricular support is based on federal funding rates, which vary widely both across NIH institutes and over time. As an inherent challenge to biomedical research funding, it is important to candidly discuss the subjective and fluctuating ecosystem of federal funding. Doing so would likely encourage trainees to distinguish between grant writing skill development from the ultimate funding decisions.

In conclusion, we believe that a structured curriculum of grant writing preparation is key to the long-term success of all MD–PhD students as they develop into independently funded physician-scientists. Regardless of whether the extramural grants they write during training are funded, the process of engaging them in grant drafting and simulated reviews offers needed insight into their academic careers.

## Data Availability

The datasets generated during and/or analyzed during the current study are available from the corresponding author on reasonable request.
